# Evaluation of the Quality of Delirium Website Content for Patient and Family Education: Cross-Sectional Study

**DOI:** 10.2196/53087

**Published:** 2025-02-20

**Authors:** Karla Krewulak, Kathryn Strayer, Natalia Jaworska, Krista Spence, Nadine Foster, Scotty Kupsch, Khara Sauro, Kirsten M Fiest

**Affiliations:** 1 Department of Critical Care Medicine University of Calgary Calgary, AB Canada; 2 Department of Community Health Sciences and O'Brien Institute for Public Health University of Calgary Calgary, AB Canada; 3 Department of Psychiatry & Hotchkiss Brain Institute University of Calgary Calgary Canada

**Keywords:** education, health information, internet, delirium, patient, caregiver, brain lesions, confusion, inattentiveness, disorientation, family education, information seeking, readability, high-quality websites, accessibility

## Abstract

**Background:**

Patients and families who have experienced delirium may seek information about delirium online, but the quality and reliability of online delirium-related websites are unknown.

**Objective:**

This study aimed to identify and evaluate online delirium-related websites that could be used for patient and family education.

**Methods:**

We searched Microsoft Bing, Google, and Yahoo using the keywords “delirium” and the misspelled “delerium” to identify delirium-related websites created to inform patients, families, and members of the public about delirium. The quality of identified delirium-related website content was evaluated by 2 authors using the validated DISCERN tool and the *JAMA* (*Journal of the American Medical Association*) benchmark criteria. Readability was assessed with the Simple Measure of Gobbledygook, the Flesch Reading Ease score, and the Flesch Kincaid grade level. Each piece of website content was assessed for its delirium-related information using a checklist of items co-designed by a working group, which included patients, families, researchers, and clinicians.

**Results:**

We identified 106 websites targeted toward patients and families, with most hospital-affiliated (21/106, 20%) from commercial websites (20/106, 19%), government-affiliated organizations (19/106, 18%), or from a foundation or advocacy group (16/106, 15%). The median time since the last content update was 3 (IQR 2-5) years. Most websites’ content (101/106, 95%) was written at a reading level higher than the recommended grade 6 level. The median DISCERN total score was 42 (IQR 33-50), with scores ranging from 20 (very poor quality) to 78 (excellent quality). The median delirium-related content score was 8 (IQR 6-9), with scores ranging from 1 to 12. Many websites lacked information on the short- and long-term outcomes of delirium as well as how common it is. The median *JAMA* benchmark score was 1 (IQR 1-3), indicating the quality of the websites’ content had poor transparency.

**Conclusions:**

We identified high-quality websites that could be used to educate patients, families, or the public about delirium. While most delirium-related website content generally meets quality standards based on DISCERN and JAMA benchmark criteria, high scores do not always ensure patient and family-friendliness. Many of the top-rated delirium content were text-heavy and complex in layout, which could be overwhelming for users seeking clear, concise information. Future efforts should prioritize the development of websites with patients and families, considering usability, accessibility, and cultural relevance to ensure they are truly effective for delirium education.

## Introduction

Delirium is a common, potentially preventable medical condition characterized by an acute onset of inattention, altered level of consciousness, or disorganized thinking. Delirium is the most common hospital-acquired complication [1-3], with greater prevalence in older adults (≥70 years of age) [1] and critically ill adults and children [2,4-7]. Emerging literature consistently highlights the negative impacts of delirium on patients (eg, increased risk of morbidity and mortality) [5,8,9] and families (eg, symptoms of distress, helplessness, and anxiety) [10-16]. Despite its prevalence and negative outcomes, delirium remains poorly recognized and is often missed by health care providers [17-19].

Families at the bedside may be important partners in delirium prevention, detection, and management. They are well-positioned to notice subtle changes in their loved one’s cognition and behavior from their prehospitalized levels and help to identify symptoms of delirium [20-23]. However, there are challenges to building effective partnerships between families and the health care team. First, families require delirium knowledge to participate in delirium care [24,25]. The literature indicates that not all health care providers engage in this aspect of patient care [26,27]. Time constraints and lack of access to sufficient educational materials limit the ability of health care providers to deliver health education on delirium [28]. Even when health care providers discuss delirium with patients and their families, the qualitative literature suggests gaps in understanding and unmet delirium information needs [29-31], which may prompt patients or families to independently seek out their own sources of delirium information.

A recent study found that families of patients admitted to an intensive care unit (ICU) had a self-reported low level of delirium knowledge and learned about delirium by searching the term online [32]. A separate study indicated families preferred obtaining delirium-related information through internet sources [24]. As families self-report accessing delirium information online, high-quality online delirium information may be one way to improve patient, family, and the public’s understanding of delirium, which can, in turn, empower them to participate in delirium prevention, detection, and management [33].

Over 10 million Americans access the internet for health information per day [34]. Recent studies report that health-related information on websites is often inaccurate, biased, misleading, or outdated [35-41]. Information about delirium is widely available on the internet, but, as with other health information on the internet, the information may be low-quality and inconsistent across sources. It is unknown if the websites of patients, families, and the public access to delirium information contain reliable, accurate, and up-to-date information.

The increasing availability of websites related to delirium is likely reflective of the creation of delirium societies or associations (American Delirium Society, European Delirium Association, and Australasian Delirium Association), World Delirium Awareness Day (established in 2017), and an increase in the implementation of regular delirium screening in hospitals [42,43]. The purpose of our study was to evaluate delirium website content based on readability, quality, and key content areas to identify high-quality delirium-related website content that could be used for patient, family, and public delirium education.

## Methods

### Website Search

**Figure 1:**
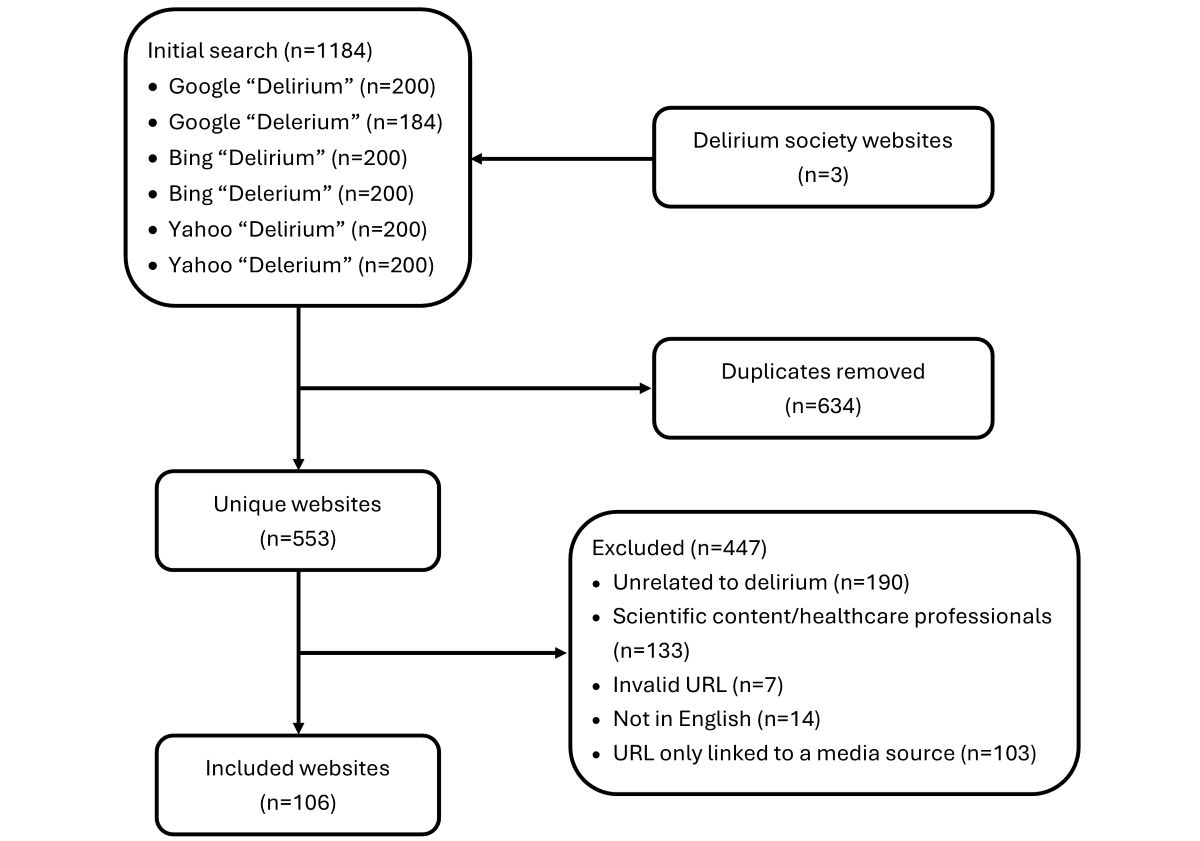
Flowchart illustrating the process of selecting websites for quality analysis.

We searched the top 3 most used search engines: Microsoft Bing [44], Google [45], and Yahoo [46-48] using the keywords “delirium” and misspelled “delerium” [49] to ensure comprehensive coverage of websites addressing the topic. “Delirium” is the primary and widely recognized term in both clinical and public domains, allowing us to retrieve the most relevant content. Including the misspelling “delerium” accounted for potential variations in user input, helping to capture additional website content that might not be optimized for the correct spelling. We disabled location identifiers, conducted the searches using a newly launched incognito or private window, and cleared cookies before each search to ensure search results were not influenced by precise geographic location or search history. We collected the top 200 search results from each search engine to capture the most relevant and widely accessed websites while maintaining consistency across engines. Search engines, such as Microsoft Bing, Google, and Yahoo, use sophisticated algorithms to rank search results based on factors such as relevance to keywords searched, content quality, and user engagement. In this study, while acknowledging that search results are typically personalized and may vary for each user based on factors such as location, search history, and preferences, for the purposes of analysis, we assumed that all users received the same search results. After removing duplicates, we excluded websites if they met any of the following exclusion criteria: (1) does not provide information on delirium, (2) written in a language other than English, (3) retrieved URL linked to a media source, (eg, YouTube channel or podcast), (4) retrieved URL targeted researchers or health care providers (eg, research articles), or (5) retrieved URL linked to a website that produced an error message, or content not available without a subscription. Despite disabling location identifiers, it is possible that search engine algorithms used IP addresses or language settings and identified the delirium association that is most closely associated with our location (ie, the American Delirium Society). As such, we also evaluated the website of the delirium association (European Delirium Association), which was not identified by the search (Figure 1).

### Website Quality

A team of patients, families, researchers, and health care providers (herein referred to as reviewers) evaluated delirium website content. The quality of website content was determined using the validated DISCERN tool (Multimedia Appendix 1) and the *JAMA* (*Journal of the American Medical Association*) benchmark criteria (Multimedia Appendix 2). DISCERN is a standardized set of criteria developed to evaluate the transparency, quality, and reliability of health information written for the public [50,51]. The DISCERN instrument has 16 questions. Each question can be scored from 1 (definite no) to 5 (definite yes). The total score can range from 16-80, wherein scores can be interpreted as excellent (63-75 points), good (51-62 points), fair (39-50 points), poor (27-38 points), and very poor (16-26 points) quality [52]. Two reviewers independently and in duplicate evaluated each website using the DISCERN instrument. DISCERN scores that differed by 5 points or more between the two reviewers were scored again by a third reviewer. The mean of all reviewers was taken as the final DISCERN score for each website. JAMA benchmark criteria include 4 standards of credible information sources: authorship (affiliations and credentials), attribution (references, sources, and copyright), disclosure (conflicts of interests), and currency (when content is posted and updated) [53]. Scores range from 0 to 4, with a score of 4 indicating the website is a credible source. Two reviewers independently and in duplicate assigned scores for each item in the JAMA benchmark criteria to indicate if it was present (score=1) or absent (score=0). Disagreements in scoring were resolved through discussion or the inclusion of a third reviewer.

### Delirium-Related Content

There are no published guidelines for evaluating delirium education materials. To evaluate the quality of content from each website, a working group of patient partners (past ICU patients and families who are members of our research team), delirium researchers, and clinicians identified seven key points that patients, families, and the public would want to know about delirium: (1) the definition of delirium (must align with definition from the *DSM-5* (*Diagnostic and Statistical Manual of Mental Disorders* Fifth Edition]) [54], the American Delirium Society, European Delirium Association, or Australasian Delirium Association [55-57]; Multimedia Appendix 3); (2) delirium risk factors [5]; (3) short and long-term outcomes of delirium [5]; (4) signs and symptoms of delirium [5,58]; (5) information to differentiate between delirium and dementia [59]; (6) delirium prevalence; and (7) strategies to prevent and manage delirium [13,15]. The DISCERN criteria evaluate treatment choices, so only items 1-6 (of 7) were evaluated. Two reviewers evaluated the websites independently and in duplicate to determine if the site provided complete and accurate information in these 6 areas (Multimedia Appendix 3)**.** Each item was scored to be present (score=2), somewhat present (ie, incorrect or incomplete information, score=1), or absent (score=0). Scores could range from 0 to 12, with a higher score indicating the website had more sufficient delirium content. Disagreements in scoring were resolved through discussion or the inclusion of a third reviewer.

### Readability

The level of difficulty of the reading material of each website was scored using the Simple Measure of Gobbledygook (SMOG) formula [60], the Flesch Reading Ease score [61], and the Flesch Kincaid grade level [62]. These scores are credible tools that are widely used to evaluate readability [63]. The included websites were evaluated if they were written at the American Medical Association and National Institutes of Health recommendation of a grade 6 reading level [64,65].

### Data Extraction and Analyses

A standardized data extraction template was created and piloted to ensure reviewers understood how to evaluate the quality, content, and readability of each website. The template included detailed column headers to ensure each website was scored the same way (eg, guidelines for rating each DISCERN question). We also collected the following variables for each website: URL or website address, date the website was created, country, free text description of the website, website creators, and patient population or clinical setting targeted. We mapped the included websites onto the patient engagement framework that described patient engagement as inform (provision of education), activate (prompts action), or collaborate (encouraging interaction with health care providers) [3]. To ensure consistency in evaluations, all reviewers underwent comprehensive training. All reviewers read the DISCERN handbook and the study protocol, which described how to evaluate the quality, content, and readability of each website. All reviewers met to go through the evaluation criteria and to evaluate one website together. We then conducted a calibration exercise wherein all reviewers independently evaluated the same 5 randomly selected websites. We met to discuss discrepancies and reached a consensus on the overall quality of the 5 websites. The remaining websites were evaluated independently and in duplicate. Quality checks were conducted at regular intervals to verify that the criteria were being interpreted and applied consistently. Reviewers met regularly to discuss progress and address any uncertainties or discrepancies identified by the quality checks.

Website scores were summarized descriptively using counts and percentages, mean (SD), or median (IQR). The Kruskal-Wallis test was used to evaluate website scores between website categories (eg, foundation or advocacy group, government, hospital, and academic institution). Statistical analyses were performed in Stata/MP (version 14.2; StataCorp LLC). A *P* value of <.05 was considered significant. We compiled the top 10 websites based on the highest weighted combined quality score. Each score-delirium-related content, readability, DISCERN, and *JAMA* benchmark was normalized using observed (readability only) or possible score ranges. Readability was reverse-scaled because a lower score is better. We weighted readability, delirium-related content, and DISCERN at 30% each, and *JAMA* benchmark criteria (website content transparency) at 10%. These weightings, determined by the working group, were used to calculate a final weighted score, where a higher score indicated higher quality.

### Ethical Considerations

As the study did not involve human participants or human biological materials, the study was deemed exempt from the ethics board approval process. This study relied on publicly available data and therefore, ethics approval was not needed for this study as per the University of Calgary Conjoint Health Research Ethics Board guidelines [66].

## Results

### Website Search

On November 21, 2024, we searched Bing, Google, and Yahoo and removed duplicates and URLs that did not meet inclusion criteria; we identified 106 websites targeted toward patients, families, and the public. This included private or public hospitals (eg, Mayo Clinic: 21/106, 20%), commercial websites (eg, Merck: 20/106, 19%), and websites affiliated with a foundation or advocacy group (eg, American Delirium Society: 16/106, 15%). A description of each website is in Multimedia Appendix 4. Over half of the included URLs (62/106, 58%) used all 3 engagement strategies: inform (eg, delirium information), activate (eg, how to prevent or manage delirium), and collaborate (eg, when or how to talk with a health care provider about delirium). A total of 17 websites (16%) used inform strategies only, and 22 websites (21%) used both inform and activate engagement strategies. Of the 82 (77%) websites that reported a date when the website was posted or updated, the median time since the last update was 3 (IQR 2-5) years.

### Website Content Quality

The median DISCERN score was 42 (IQR 33-50), representing fair quality. The quality ranged from 20 (very poor quality) to 78 (excellent quality). Many websites did not include a list of the sources used to compile the information on the website, refer to areas of uncertainty (eg, delirium may be missed or difficult to identify, no guideline-recommended pharmacological treatments for delirium), nor describe risks of each treatment (eg, antipsychotics if used for severe agitation). Several websites shared information that was not backed by a current evidence synthesis [5] or available guidelines [13,15]. This included claims that antipsychotics were the first line of treatment for delirium without discussion of their risk of prolonging or exacerbating delirium symptoms. Many websites mention antipsychotics as a means to manage the symptoms of delirium, such as agitation, which is a safety concern (supported by recent guidelines) [13,15]. DISCERN scores were significantly different between website categories (ie, academic, commercial, foundation or advocacy group, etc).

The median JAMA score was 1 (IQR 1-3), with 17/106 websites (16%) scoring 0, which indicates most websites lacked transparency. In particular, most websites did not list authors or contributors, their affiliations, and relevant credentials (67/106, 63%) nor included references and sources for all content (65/106, 61%). JAMA scores were not significantly different between website categories (ie, academic, commercial, foundation or advocacy group, etc). The median scores for delirium website transparency can be seen in Table 1**.**

**Table 1:** Comparison of the median DISCERN, Journal of the American Medical Association (JAMA) benchmark score, and weighted quality scores across website categories.

Website category	Websites, n (%)	DISCERN score (range 16-80), median (IQR)	JAMA benchmark score (range 0-4), median (IQR)	Weighted quality score (range 0-100), median (IQR)
Hospital affiliated	21 (19.8)	37.5 (32-44.5)	1 (0.8-3)	55.9 (52.4-59.4)
Commercial	20 (18.9)	44.5 (34.5-52.3)	3 (2-4)	57.4 (49.4-66.6)
Government	19 (17.9)	42 (32.5-51.5)	1 (1-2)	54.5 (47-64.3)
Foundation or advocacy organization	16 (15)	46.5(42.5-50.3)	1 (0-1.3)	58.4 (55.7-65.7)
Regional health authority	14 (13.2)	32.5 (27.3-40.5)	1 (1-1.8)	49.4 (46.1-56.2)
Academic	6 (5.7)	52.5 (50-57.3)	1.5 (1-2)	64.4 (60.2-70.2)
General reference or educational resource	6 (5.7)	40.5 (32.8-55)	2.5 (2-3)	54.3 (52.5-63.5)
Professional organization	4 (3.8)	34.0 (33-38)	1.5 (1-2)	50.1 (41.3-57.4)

### Delirium-Related Content

The median score for delirium-related content was 8 (IQR 6-9). Nearly half of the websites included a complete definition of delirium (77/106, 73%). Website evaluators rated several websites (26/106, 25%) to have a somewhat correct definition of delirium (eg, missing items from the *DSM-5* or delirium society definitions of delirium). A majority of the websites (77/106, 73%) included predisposing (eg, age and dementia) and precipitating delirium risk factors (eg, surgery and dehydration) that are consistent with available evidence [5]. Websites that reported somewhat correct delirium risk factors (23/106, 22%) reported incomplete or inaccurate risk factors. These websites either focused solely on predisposing or precipitating factors, overlooked key groups like infants and young children, or included risk factors not widely associated with delirium in the literature (eg, male sex). Most websites (83/106, 78%) included the signs and symptoms of delirium. Those websites that somewhat described the signs and symptoms of delirium (19/106, 18%) often missed a hallmark of delirium (eg, inattention, acute onset, fluctuating course, or disorganized thinking). Many websites did not include short and long-term outcomes associated with delirium (54/106, 51%). This included not describing delirium as a risk factor for dementia or the association between delirium and risk for long-term cognitive decline. Many websites did not describe the prevalence of delirium (41/106, 39%) or vaguely described its prevalence (eg, “common;” 31/106, 29%). Half of the websites stated that there was a difference between dementia and delirium (54/106, 51%). Out of these 54 websites, 43 (80%) described in detail how dementia and delirium differed. Table 2 shows the mean scores for delirium content.

**Table 2:** Comparison of the mean delirium content scores across website categories.

Website category	Website, n (%)	Delirium content^a^					
		Delirium definition, mean (SD)	Risk factors and causes, mean (SD)	Short- and long-term outcomes, mean (SD)	Signs and symptoms, mean (SD)	Differences between delirium and dementia, mean (SD)	Delirium prevalence, mean (SD)
Hospital affiliated	21 (19.8)	1.6 (0.6)	1.5 (0.6)	0.7 (0.7)	1.6 (0.5)	0.6 (0.9)	0.9 (0.8)
Commercial	20 (18.9)	1.6 (0.5)	1.9 (0.4)	0.7 (0.9)	1.9 (0.5)	1.2 (0.9)	1 (0.9)
Government	19 (17.9)	1.9 (0.4)	1.9 (0.3)	0.5 (0.7)	1.9 (0.2)	0.9 (1.0)	0.8 (0.9)
Foundation or advocacy organization	16 (15)	1.8 (0.4)	1.7 (0.5)	0.9 (0.9)	1.9 (0.2)	1.3 (0.8)	0.8 (0.7)
Regional health authority	14 (13.2)	1.9 (0.3)	1.4 (0.8)	0.7 (0.8)	1.6 (0.6)	0.8 (0.9)	0.8 (0.9)
Academic	6 (5.7)	2 (0)	1.5 (0.5)	1.2 (0.7)	2 (0)	1.2 (0.9)	1.7 (0.5)
General reference or educational resource	6 (5.7)	1.3 (0.7)	1.7 (0.7)	1.3 (0.9)	1.3 (0.7)	0.7 (0.9)	1.3 (0.7)
Professional organization	4 (3.8)	1.3 (0.4)	1.5 (0.9)	0.3 (0.4)	1.3 (0.8)	0.5 (0.9)	1 (0.7)

^a^For all criteria, the minimum possible score=0, maximum=2.

### Readability

The median SMOG readability score was a grade 15 (IQR 13-17, range 10-22) level. The median Flesch Kincaid Reading Level score was a grade 10 (IQR 9-12, range 5-21) level. The median Flesch Reading Ease score was 47 (IQR 38-56; range 0-80.7), indicating the websites are difficult to read for most of the population. Of the 106 websites, 5 (5%) were written at a reading level equal to or lower than grade 6.

### Top 10 Websites for Patient, Family, and Public Information on Delirium

The median for the weighted quality score for websites was 56.1 (IQR 49.3-65.5, range 28.8-84.1). Based on the normalized and weighted evaluation criteria, the top 10 websites for delirium for patient and family education are summarized in Table 3. The Mayo Clinic website, which appeared as the top result for Google and Yahoo “delirium” searches, ranked 15th overall based on weighted scores for quality, content, and readability, with an overall weighted score similar to the top 10 websites (69.8). In contrast, one of the websites listed among the top 10 search results ranked 83rd overall due to incomplete and low-quality information such as inaccurate claims (eg, counseling as a method to address disorientation, male sex as a risk factor, and a section on “confusion.”). The Wikipedia entry on delirium was in the first 10 results of Microsoft Bing (3rd), Google (5th), and Yahoo (4th). However, it had an overall weighted score of 54.8 due to its poor readability. Weighted quality scores were not significantly different between website categories (ie, academic, commercial, foundation or advocacy group, etc).

**Table 3:** The top 10 websites ranked by weighted, combined quality, delirium content, and readability scores.

Website^a^	Position in the Bing, Google, and Yahoo search				DISCERN score (range 16-80)		Flesh Kincaid Grade Level		Delirium content score (range 0-12)		Composite weighted score (rank)
	Bing	Google	Yahoo								
Healthline.com [67]	92	7	69	75		10.5		11		84.1 (1)	
Aarp.org [68]	—^b^	127	—	77		10.7		11		82.6 (2)	
Sign.ac.uk [69]	—	108	—	59		8.3		12		78.2 (3)	
UpToDate.com [70]	—	14	—	64		12.9		12		76.9 (4)	
HRH.ca [71]	—	96	—	64		9.3		9		73.7 (5)	
RGPToronto.ca [72]	106	55	112	78		12.3		10		73.5 (6)	
Merckmanuals.com [73]	—	12	180	53		12.4		12		72.7 (7)	
Healthify.nz [74]	—	99	—	38		8		9		72.7 (8)	
Verywellhealth.com [75]	142	94	9	52		12.2		12		72.6 (9)	
Clevelandclinic.org [76]	1	2	2	62		9.6		9		72.3 (10)	

^a^All websites listed, including UpToDate (which typically requires a subscription), are publicly available.

^b^Indicates the website was not in the top 200 of search results for that search engine.

## Discussion

### Principal Findings

Delirium websites are one source of delirium education to prepare families to partner with delirium prevention, detection, and management. This study reports on the quality, content, and readability of websites with delirium information for patients, families, and the public. Overall, our findings suggest the quality of delirium-related website content is fair, with many websites lacking credibility and transparency. The American Medical Association and National Institutes of Health recommend that patient education materials be written at a grade 6 reading level [64,65], but nearly all websites (100/106, 95%) were written at higher than a grade 6 reading level. Furthermore, websites that families may encounter when looking for information about delirium may include incomplete information about delirium, overlook key groups such as infants or young children, or provide outdated or inaccurate information. This study identified the 10 best websites that patients, families, and clinicians can refer to, to find information about delirium.

It is crucial that high-quality and easily understandable websites on delirium are available to patients, families, and the public for several reasons. First, delirium is a common and serious medical condition that can be confusing and frightening for patients and families [4,10-13,77,78]. This may prompt families to search for delirium on the internet. Clear and concise information will help families to better understand delirium, its causes, signs, and prevention or management options. Second, countries and organizations promote engaging families with patient care [16,23,79]. To be active participants in delirium care, families must be provided with delirium education. Accessible health information can empower patients and families to participate in delirium prevention, detection, and management [20,80] and seek medical attention when necessary. Like any family engagement intervention, not all families may want to participate in delirium care, and they have reported that one of their preferential ways to receive information is through the internet. As such, websites should offer information that informs (providing information on delirium), activates (encouraging families to prevent and manage delirium), and collaborates (preparing families to discuss delirium with health care teams) with families to cater to a broad audience with varying information needs.

To improve the quality of delirium websites, it is essential to identify common deficiencies among them. A majority of the included websites did not provide proper citations for the information used in the website content. Including sources on websites benefits readers by fostering credibility, transparency, and accountability of the website information and enables readers to access additional delirium resources. Most websites (100/106, 95%) were written above the recommended grade 6 level. It is important that health information is presented in a manner that is accessible to its target so that the information is not misinterpreted and to enable the reader to make informed decisions about their health. Other studies evaluating the quality of health information on websites also report that health information is not written at an appropriate reading level [81-85]. While website creators can modify their content to meet the grade 6 recommendation, the best practice would be to codevelop websites with patients, families, and the public. Websites should also include the following delirium-related content: (1) the definition of delirium (from the *DSM-5* [54] or from delirium societies or associations) [55-57], (2) delirium risk factors [5], (3) short and long-term outcomes of delirium [5], (4) signs and symptoms of delirium [54,58], (5) information to differentiate between delirium and dementia [59], (6) delirium prevalence [1,2,4-7], and (7) strategies to prevent and manage delirium [13,15]. With more people using the internet to access health information, it is imperative that website developers follow the above guidance to ensure websites include the highest quality and readable delirium information for patients, families, and the public.

This study has several implications for practice. First, there is a need for families to be integrated as partners in delirium care [86]. An important first step is to provide families with delirium information to prepare them to participate in delirium prevention, detection, and management. However, staff often lack the time to provide comprehensive delirium education to families due to heavy workloads and clinical responsibilities. Health care providers can leverage this curated list of websites to supplement delirium information provided to patients and families. By directing families to these websites, they can ensure families are consulting the highest quality and most reliable delirium information currently available on the internet. Second, it is clear that higher-quality delirium websites with plain language are needed. This can be accomplished by hospital organizations or delirium societies creating their own delirium education materials. Hospitals can leverage the set of criteria (ie, DISCERN, JAMA benchmark, readability measures, and delirium content) when adapting or creating delirium-related materials on their websites. Furthermore, there remains a need for policymakers to prioritize the importance of digital health literacy. This might include initiatives to enhance how people navigate health information on websites. This might also include advocating for high-quality educational resources.

### Strengths and Limitations

There are several strengths and limitations that should be considered in this study. To our knowledge, this is the first study to identify and evaluate websites on delirium that could be used for patient and family education. Delirium websites were identified using the top 3 most used search engines (Bing, Google, and Yahoo). Finally, all study activities included patient and family partners. Despite these strengths, there are several limitations that should be considered. First, the search strategy consisted of only 2 search terms: delirium and the most commonly misspelled form of delirium (delerium). It is possible that patients, families, and the public may use other terms to search for delirium information, and, as such, some websites may have been missed. Second, the search was performed in Canada. Despite disabling location services, which limited the extent to which the search engines can use our location, other aspects of our location may have been inferred based on our IP address or language settings. For this reason, and that our team was proficient in English, we only included websites written in English. As such, the results of this study may not be generalizable to people who do not speak English. To attain a more comprehensive understanding of delirium-related websites in languages other than English, further research is warranted. This should include other prominent search engines with significant market shares (eg, Yandex, Baidu, Petal Search, and DuckDuckGo) and collaborative efforts with researchers from diverse linguistic backgrounds and countries. Third, the analysis of the websites was limited to what was reported, which may not have comprehensively captured the development of the website. This may include if websites used credible sources or delirium experts to compile the websites but did not cite the sources or identify the delirium experts. Finally, the tool that was used to evaluate delirium content has not been externally validated, as it was developed specifically for this study. Finally, while DISCERN, JAMA benchmark, delirium content, and readability tools are robust for assessing quality and transparency, they focus on structural elements rather than usability or accessibility for patients and families. Websites with high scores often had dense information or busy layouts that may overwhelm patients or families seeking concise and accessible information. These metrics do not account for patient or family experience, engagement, or cultural relevance, which are critical for effective patient and family education.

### Conclusion

This study suggests delirium websites for the public are of fair quality. Inadequacies in evaluated websites, such as lack of transparency, incomplete delirium information, and poor readability, should be addressed when updating current or creating new delirium websites aimed at patients, families, or the public. Following the outlined standards for quality, delirium content, and readability will ensure high-quality, transparent, and accessible delirium information for patients, families, and the public.

## Appendix

Multimedia Appendix 1 The DISCERN instrument.

Multimedia Appendix 2 JAMA (Journal of the American Medical Association) benchmark criteria for credibility of medical information on the internet.

Multimedia Appendix 3 Evaluation of Delirium Content Guide.

Multimedia Appendix 4 Description of the websites included in the study.

## Abbreviations

DSM-5: Diagnostic and Statistical Manual of Mental Disorders (Fifth Edition)
ICU: intensive care unit
JAMA: Journal of the American Medical Association
SMOG: Simple Measure of Gobbledygook

## Data Availability

The datasets generated during this study are available from the corresponding author on reasonable request.

## References

[1] Gibb K, Seeley A, Quinn T, Siddiqi N, Shenkin S, Rockwood K, Davis D (2020). The consistent burden in published estimates of delirium occurrence in medical inpatients over four decades: a systematic review and meta-analysis study. Age Ageing.

[2] Krewulak KD, Stelfox HT, Leigh JP, Ely EW, Fiest KM (2018). Incidence and prevalence of delirium subtypes in an adult ICU: a systematic review and meta-analysis. Crit Care Med.

[3] Fiest KM, McIntosh CJ, Demiantschuk D, Leigh JP, Stelfox HT (2018). Translating evidence to patient care through caregivers: a systematic review of caregiver-mediated interventions. BMC Med.

[4] Ely EW, Inouye SK, Bernard GR, Gordon S, Francis J, May L, Truman B, Speroff T, Gautam S, Margolin R, Hart RP, Dittus R (2001). Delirium in mechanically ventilated patients: validity and reliability of the confusion assessment method for the intensive care unit (CAM-ICU). JAMA.

[5] Wilson JE, Mart MF, Cunningham C, Shehabi Y, Girard TD, MacLullich AMJ, Slooter AJC, Ely EW (2020). Delirium. Nat Rev Dis Primers.

[6] Traube C, Silver G, Reeder R, Doyle H, Hegel E, Wolfe H, Schneller C, Chung MG, Dervan LA, DiGennaro JL, Buttram SDW, Kudchadkar SR, Madden K, Hartman ME, deAlmeida ML, Walson K, Ista E, Baarslag MA, Salonia R, Beca J, Long D, Kawai Yu, Cheifetz IM, Gelvez J, Truemper EJ, Smith RL, Peters ME, O'Meara AMIqbal, Murphy S, Bokhary A, Greenwald BM, Bell MJ (2017). Delirium in critically Ill children: an international point prevalence study. Crit Care Med.

[7] Semple D, Howlett M, Strawbridge J, Breatnach C, Hayden J (2022). A systematic review and pooled prevalence of delirium in critically ill children. Crit Care Med.

[8] Witlox J, Eurelings LSM, de Jonghe JFM, Kalisvaart KJ, Eikelenboom P, van Gool WA (2010). Delirium in elderly patients and the risk of postdischarge mortality, institutionalization, and dementia: a meta-analysis. JAMA.

[9] Cole MG, Bailey R, Bonnycastle M, McCusker J, Fung S, Ciampi A, Belzile E, Bai C (2015). Partial and no recovery from delirium in older hospitalized adults: frequency and baseline risk factors. J Am Geriatr Soc.

[10] Williams ST, Dhesi JK, Partridge JSL (2020). Distress in delirium: causes, assessment and management. Eur Geriatr Med.

[11] Luth EA, Maciejewski PK, Phongtankuel V, Xu J, Prigerson HG (2021). Associations between hospice care and scary family caregiver experiences. J Pain Symptom Manage.

[12] Partridge JSL, Martin FC, Harari D, Dhesi JK (2013). The delirium experience: what is the effect on patients, relatives and staff and what can be done to modify this?. Int J Geriatr Psychiatry.

[13] (2023). Delirium: Prevention, Diagnosis and Management in Hospital and Long-Term Care.

[14] Davis D, Searle S, Tsui A (2019). The Scottish intercollegiate guidelines network: risk reduction and management of delirium. Age Ageing.

[15] Devlin JW, Skrobik Y, Gélinas C, Needham DM, Slooter AJC, Pandharipande PP, Watson PL, Weinhouse GL, Nunnally ME, Rochwerg B, Balas MC, van den Boogaard M, Bosma KJ, Brummel NE, Chanques G, Denehy L, Drouot X, Fraser GL, Harris JE, Joffe AM, Kho ME, Kress JP, Lanphere JA, McKinley S, Neufeld KJ, Pisani MA, Payen J, Pun BT, Puntillo KA, Riker RR, Robinson BRH, Shehabi Y, Szumita PM, Winkelman C, Centofanti JE, Price C, Nikayin S, Misak CJ, Flood PD, Kiedrowski K, Alhazzani W (2018). Clinical practice guidelines for the prevention and management of pain, agitation/sedation, delirium, immobility, and sleep disruption in adult patients in the ICU. Crit Care Med.

[16] O'Rourke M (2007). he Australian commission on safety and quality in health care agenda for improvement and implementation. Asia Pacific Journal of Health Management.

[17] Inouye SK, Westendorp RG, Saczynski JS (2014). Delirium in elderly people. Lancet.

[18] Geriatric Medicine Research Collaborative (2019). Delirium is prevalent in older hospital inpatients and associated with adverse outcomes: results of a prospective multi-centre study on world delirium awareness day. BMC Med.

[19] Lange PW, Lamanna M, Watson R, Maier AB (2019). Undiagnosed delirium is frequent and difficult to predict: results from a prevalence survey of a tertiary hospital. J Clin Nurs.

[20] Fiest KM, Krewulak KD, Ely EW, Davidson JE, Ismail Z, Sept BG, Stelfox HT (2020). Partnering with family members to detect delirium in critically ill patients. Crit Care Med.

[21] Steis MR, Evans L, Hirschman KB, Hanlon A, Fick DM, Flanagan N, Inouye SK (2012). Screening for delirium using family caregivers: convergent validity of the family confusion assessment method and interviewer-rated confusion assessment method. J Am Geriatr Soc.

[22] Shulman RW, Kalra S, Jiang JZ (2016). Validation of the sour seven questionnaire for screening delirium in hospitalized seniors by informal caregivers and untrained nurses. BMC Geriatr.

[23] Aggar C, Craswell A, Bail K, Compton R, Hughes M, Sorwar G, Baker J, Shinners L, Greenhill J (2023). Partnering with carers in the management of delirium in general acute care settings: an integrative review. Australas J Ageing.

[24] Bull MJ, Boaz L, Sjostedt JM (2016). Family caregivers' knowledge of delirium and preferred modalities for receipt of information. J Appl Gerontol.

[25] Tonna JE (2020). Negative studies should inform our research and care: engaging family members in the care of the critically Ill. Crit Care Med.

[26] Smithburger PL, Korenoski AS, Kane-Gill SL, Alexander SA (2017). Perceptions of family members, nurses, and physicians on involving patients' families in delirium prevention. Crit Care Nurse.

[27] Cohen C, Pereira F, Kampel T, Bélanger L (2021). Integration of family caregivers in delirium prevention care for hospitalized older adults: a case study analysis. J Adv Nurs.

[28] Kääriäinen M, Kyngäs H (2010). The quality of patient education evaluated by the health personnel. Scand J Caring Sci.

[29] Bohart S, Merete Møller A, Forsyth Herling S (2019). Do health care professionals worry about delirium? Relatives' experience of delirium in the intensive care unit: a qualitative interview study. Intensive Crit Care Nurs.

[30] Toye C, Matthews A, Hill A, Maher S (2014). Experiences, understandings and support needs of family carers of older patients with delirium: a descriptive mixed methods study in a hospital delirium unit. Int J Older People Nurs.

[31] Schmitt EM, Gallagher J, Albuquerque A, Tabloski P, Lee HJ, Gleason L, Weiner LS, Marcantonio ER, Jones RN, Inouye SK, Schulman-Green D (2019). Perspectives on the delirium experience and its burden: common themes among older patients, their family caregivers, and nurses. Gerontologist.

[32] Parsons Leigh J, Krewulak KD, Zepeda N, Farrier CE, Spence KL, Davidson JE, Stelfox HT, Fiest KM (2021). Patients, family members and providers perceive family-administered delirium detection tools in the adult ICU as feasible and of value to patient care and family member coping: a qualitative focus group study. Can J Anaesth.

[33] Lee J, Yeom I, Yoo S, Hong S (2023). Educational intervention for family caregivers of older adults with delirium: an integrative review. J Clin Nurs.

[34] Fox M, Duggan M (2013). Health Online 2013 Pew Research Center.

[35] Dornan BA, Oermann MH (2006). Evaluation of breastfeeding web sites for patient education. MCN Am J Matern Child Nurs.

[36] Nichols C, Oermann MH (2005). An evaluation of bariatric Web sites for patient education and guidance. Gastroenterol Nurs.

[37] Oermann M, McInerney SM (2007). An evaluation of sepsis web sites for patient and family education. Plast Surg Nurs.

[38] Oermann MH, Gerich J, Ostosh L, Zaleski S (2003). Evaluation of asthma websites for patient and parent education. J Pediatr Nurs.

[39] Oermann MH, Lowery NF, Thornley J (2003). Evaluation of web sites on management of pain in children. Pain Manag Nurs.

[40] Labovitch RS, Bozic KJ, Hansen E (2006). An evaluation of information available on the internet regarding minimally invasive hip arthroplasty. J Arthroplasty.

[41] Eysenbach G, Powell J, Kuss O, Sa ER (2002). Empirical studies assessing the quality of health information for consumers on the world wide web: a systematic review. JAMA.

[42] Bauernfreund Y, Butler M, Ragavan S, Sampson EL (2018). TIME to think about delirium: improving detection and management on the acute medical unit. BMJ Open Qual.

[43] Martin L, Lyons M, Patton A, O Driscoll M, McLoughlin K, Hannon E, Deasy C (2022). Implementing delirium screening in the emergency department: a quality improvement project. BMJ Open Qual.

[44] Bing. Microsoft.

[45] Google. Google Search n.d.

[46] (2021). Top 8 best search engines (of 2021). RapidApi.

[47] (2020). Meet the top 10 search engines in the world in 2021. Oberlo.

[48] Yahoo search. Yahoo.

[49] Reddin C, Koay WJ, Mulkerrin EC, OʼKeeffe ST (2020). Misspelling of delirium as "Delerium" in the academic literature. J Am Geriatr Soc.

[50] Charnock D, Shepperd S (2004). Learning to DISCERN online: applying an appraisal tool to health websites in a workshop setting. Health Educ Res.

[51] (1997). The DISCERN instrument. DISCERN Online.

[52] Weil AG, Bojanowski MW, Jamart J, Gustin T, Lévêque M (2014). Evaluation of the quality of information on the Internet available to patients undergoing cervical spine surgery. World Neurosurg.

[53] Silberg WM, Lundberg G D, Musacchio R A (1997). Assessing, controlling, and assuring the quality of medical information on the Internet: Caveant lector et viewor--Let the reader and viewer beware. JAMA.

[54] (2013). Diagnostic and Statistical Manual of Mental Disorders 5th ed (DSM-5).

[55] American Delirium Society.

[56] European Delirium Association, American Delirium Society (2014). The DSM-5 criteria, level of arousal and delirium diagnosis: inclusiveness is safer. BMC Med.

[57] Australasian Delirium Association.

[58] Inouye SK, van Dyck CH, Alessi CA, Balkin S, Siegal AP, Horwitz RI (1990). Clarifying confusion: the confusion assessment method. A new method for detection of delirium. Ann Intern Med.

[59] Forrant JA (2009). Dementia or delirium: do you know the difference?. Nurs Crit Care.

[60] McLaughlin GH (1969). SMOG grading: a new readability formula. Journal of Reading.

[61] Flesch R (1948). A new readability yardstick. J Appl Psychol.

[62] Kincaid J, Fishburne R, Rogers R, Chissom B Derivation of new readability formula for navy enlisted personnel.

[63] Friedman DB, Hoffman-Goetz L (2006). A systematic review of readability and comprehension instruments used for print and web-based cancer information. Health Educ Behav.

[64] Weiss BD (2003). Health Literacy.

[65] MedlinePlus: how to write easy to read health materials. National Institutes of Health.

[66] Resources: Ethics and Compliance. University of Calgary.

[67] Healthline.

[68] AARP.

[69] SIGN.

[70] UpToDate.

[71] Humber River Health.

[72] RGP Toronto.

[73] Merck manuals.

[74] Healthify.

[75] verywell health.

[76] Cleveland Clinic.

[77] Siddiqi N, House AO, Holmes JD (2006). Occurrence and outcome of delirium in medical in-patients: a systematic literature review. Age Ageing.

[78] Almeida ICT, Soares M, Bozza FA, Shinotsuka CR, Bujokas R, Souza-Dantas VC, Ely EW, Salluh JIF (2014). The impact of acute brain dysfunction in the outcomes of mechanically ventilated cancer patients. PLoS One.

[79] Davidson JE, Zisook S (2017). Implementing family-centered care through facilitated sensemaking. AACN Adv Crit Care.

[80] Determeijer JJ, Leopold SJ, Spijker R, Agyemang C, van Vugt M (2023). Family participation to enhance care and tackle health worker shortages in resource-limited hospitals: a systematic review. J Glob Health.

[81] Kaicker J, Dang W (2013). Assessing the quality and reliability Of health information on ERCP using the DISCERN instrument. Health Care: Current Reviews.

[82] Saleh D, Fisher JH, Provencher S, Liang Z, Ryerson CJ, Weatherald J (2022). A systematic evaluation of the quality, accuracy, and reliability of internet websites about pulmonary arterial hypertension. Ann Am Thorac Soc.

[83] Corcelles R, Daigle CR, Talamas HR, Brethauer SA, Schauer PR (2015). Assessment of the quality of Internet information on sleeve gastrectomy. Surg Obes Relat Dis.

[84] Roughead T, Sewell D, Ryerson CJ, Fisher JH, Flexman AM (2016). Internet-based resources frequently provide inaccurate and out-of-date recommendations on preoperative fasting: a systematic review. Anesth Analg.

[85] Guo WJ, Wang WK, Xu D, Qiao Z, Shi YL, Luo P (2019). Evaluating the quality, content, and readability of online resources for failed back spinal surgery. Spine (Phila Pa 1976).

[86] Aggar C, Craswell A, Bail K, Compton RM, Hamiduzzaman K, Sorwar G, Hughes M, Greenhill J, Shinners L, Baker JR (2022). Commentary: prevention and management of delirium in older Australians: the need for the integration of carers as partners in care. Lancet Reg Health West Pac.

