# Supplementary material for: Evaluation of the Quality of Delirium Website Content for Patient and Family Education: Cross-Sectional Study
Source: J Med Internet Res. 2025 Feb 20;27:e53087. doi: 10.2196/53087 (PMC11888015; doi:10.2196/53087)
Supplement: Multimedia Appendix 1 [file jmir_v27i1e53087_app1.docx]

This is a multimedia Appendix to a full manuscript published in the J Med Internet Res. For full copyright and citation information see http://dx.doi.org/10.2196/jmir.xxxx

**Appendix 1.** The DISCERN Instrument

|  | **No** |  | **Partially** |  | **Yes** |
| --- | --- | --- | --- | --- | --- |
| *Scoring* | 1 | 2 | 3 | 4 | 5 |
| **Section 1: Is the publication reliable?** |  |  |  |  |  |
| 1. Are the aims clear?   *Hint: Look for a clear indication at the beginning of the publication of:*   - *What it is about* - *What it is meant to cover (and what tops are meant to be excluded)* - *Who might find it useful*   If the answer to Question 1 is “No,” go directly to Question 3 |  |  |  |  |  |
| 1. Does it achieve its aims?   *Hint: Consider whether the publication proves the information it aimed to as outlined in Question 1* |  |  |  |  |  |
| 1. Is it relevant?   *Hint: Consider whether:*   - *The publication addresses the questions that readers might ask* - *Recommendations and suggestions concerning treatment choices are realistic or appropriate* |  |  |  |  |  |
| 1. Is it clear what sources of information were used to compile the publication (other than the author or producer)?   *Hint:*   - Check whether the main claims or statements made about treatment choices are accompanied by a reference to the sources used as evidence, e.g. a research study or expert opinion. - Look for a means of checking the sources used such as a bibliography/reference list or the addresses of the experts or organisations quoted, or external links to the online sources.   Rating note: In order to score a full '5' the publication should fulfil both hints. Lists of additional sources of support and information (Question 7) are not necessarily sources of evidence for the current publication. |  |  |  |  |  |
| 1. Is it clear when the information used or reported in the publication was produced?   *Hint*: Look for:   - Dates of the main sources of information used to compile the publication - Date of any revisions of the publication (but not dates of reprinting in the case of print publications) - Date of publication (copyright date).   Rating note: The hints are placed in order of importance - in order to score a full '5' the dates relating to the first hint should be found. |  |  |  |  |  |
| 1. Is it balanced and unbiased?   *Hint*: Look for:   - A clear indication of whether the publication is written from a personal or objective point of view - Evidence that a range of sources of information was used to compile the publication, e.g. more than one research study or expert - Evidence of an external assessment of the publication.   Be wary if:   - The publication focuses on the advantages or disadvantages of one particular treatment choice without reference to other possible choices - The publication relies primarily on evidence from single cases (which may not be typical of people with this condition or of responses to a particular treatment) - The information is presented in a sensational, emotive or alarmist way. |  |  |  |  |  |
| 1. Does it provide details of additional sources of support and information?   *Hint:* Look for suggestions for further reading or for details of other organisations providing advice and information about the condition and treatment choices. |  |  |  |  |  |
| 1. Does it refer to areas of uncertainty?   *Hint:* Look for discussion of the gaps in knowledge or differences in expert opinion concerning treatment choices.  Be wary if the publication implies that a treatment choice affects everyone in the same way, e.g. 100% success rate with a particular treatment. |  |  |  |  |  |
| **Section 2: How good is the quality of information on treatment choices?**  N.B. The questions apply to the treatment (or treatments) described in the publication. Self-care is considered a form of treatment throughout this section. |  |  |  |  |  |
| 1. Does it describe how each treatment works?   *Hint:* Look for a description of how a treatment acts on the body to achieve its effect. |  |  |  |  |  |
| 1. Does it describe the benefits of each treatment?   *Hint:* Benefits can include controlling or getting rid of symptoms, preventing recurrence of the condition and eliminating the condition, both short-term and long-term. |  |  |  |  |  |
| 1. Does it describe the risks of each treatment?   *Hint:* Risks can include side-effects, complications and adverse reactions to treatment, both short-term and long-term. |  |  |  |  |  |
| 1. Does it describe what would happen if no treatment is used?   *Hint:* Look for a description of the risks and benefits of postponing treatment, of watchful waiting (i.e. monitoring how the condition progresses without treatment) or of permanently forgoing treatment. |  |  |  |  |  |
| 1. Does it describe how the treatment choices affect overall quality of life?   *Hint: Look for:*   - *Description of the effects of the treatment choices on day-to-day activity* - *Description of the effects of the treatment choices on relationships with family, friends and carers.* |  |  |  |  |  |
| 1. Is it clear that there may be more than one possible treatment choice?   *Hint:* Look for:   - A description of who is most likely to benefit from each treatment choice mentioned, and under what circumstances - Suggestions of alternatives to consider or investigate further (including choices not fully described in the publication) before deciding whether to select or reject a particular treatment choice. |  |  |  |  |  |
| 1. Does it provide support for shared decision making?   *Hint:* Look for suggestions of things to discuss with family, friends, doctors or other health professionals concerning treatment choices. |  |  |  |  |  |
| **Section 3: Overall rating of the publication** | **Low** |  | **Moderate** |  | **High** |
| 1. Based on the answers to all of the above questions, rate the overall quality of the publication as a source of information about treatment choices | *Serious or extensive shortcomings* |  | *Potentially important but not serious shortcomings* |  | *Minimal shortcomings* |
|  | 1 | 2 | 3 | 4 | 5 |
