# Supplementary material for: Evaluation of the Quality of Delirium Website Content for Patient and Family Education: Cross-Sectional Study
Source: J Med Internet Res. 2025 Feb 20;27:e53087. doi: 10.2196/53087 (PMC11888015; doi:10.2196/53087)
Supplement: Multimedia Appendix 2 [file jmir_v27i1e53087_app2.docx]

This is a multimedia Appendix to a full manuscript published in the J Med Internet Res. For full copyright and citation information see http://dx.doi.org/10.2196/jmir.xxxx

**Appendix 2.** Journal of the American Medical Association (JAMA) benchmark criteria for credibility of medical information on the internet.

1. *Authorship :* Authors and contributors, their affiliations, and relevant credentials should be provided.
2. *Attribution*: References and sources for all content should be listed clearly, and all relevant copyright information noted.
3. *Disclosure*: Website "ownership" should be prominently and fully disclosed, as should any sponsorship, advertising, underwriting, commercial funding arrangements or support, or potential conflicts of interest. This includes arrangements in which links to other sites are posted as a result of financial considerations. Similar standards should hold in discussion forums.
4. *Currency*: Dates that content was posted and updated should be indicated.
