# Supplementary material for: Evaluation of the Quality of Delirium Website Content for Patient and Family Education: Cross-Sectional Study
Source: J Med Internet Res. 2025 Feb 20;27:e53087. doi: 10.2196/53087 (PMC11888015; doi:10.2196/53087)
Supplement: Multimedia Appendix 3 [file jmir_v27i1e53087_app3.docx]

This is a multimedia Appendix to a full manuscript published in the J Med Internet Res. For full copyright and citation information see http://dx.doi.org/10.2196/jmir.xxxx

**Appendix 3.** Evaluation of Delirium Content Guide

| **Question** | **No (score=0)** | **Somewhat (score=1)** | **Yes (score=2)** |
| --- | --- | --- | --- |
| 1. Does the website include a definition of delirium?   **DSM-5:**  The DSM-5 defines delirium as a neurocognitive disorder characterized by a disturbance in attention (e.g., reduced ability to direct, focus, sustain, and shift attention), awareness, or cognition (e.g., memory deficit, disorientation, language, or perception) that develops over a short period of time (usually hours to a few days) and may fluctuate in severity throughout the course of a day. These disturbances are not explained by other pre-existing conditions or established disorders, and do not occur in the context of severely reduced level of arousal, such as coma1, 2.  **American Delirium Society:**  “Delirium is a state of confusion that comes on very suddenly and lasts hours to days. If your loved one becomes delirious, it means she/he cannot think very clearly, can’t pay attention and is not really aware of their environment. Sometimes people will use phrases such as “a change in mental status”, “sundowning” or “ICU psychosis”, but the precise medical term is delirium3.”  **European Delirium Association:**  “Delirium is a sign that someone is physically unwell.  It is a SUDDEN change over a few hours or days and tends to vary at different times of day. People may be confused at some times and then seem their normal selves at other times.  People who become delirious may start behaving in ways that are unusual for them- they may become more agitated than normal or feel sleepier and more withdrawn.  It may be more difficult to hold a conversation with them or they may ramble and jump from one topic to another. They may not know where they are or think they are somewhere completely different, on holiday for example.  Sometimes people who are delirious may see things that other people can’t see, or believe things that are not true.”    **Australasian Delirium Association:**  “Delirium causes changes to the person’s usual mental functioning. Its onset is rapid (hours or days). It is sometimes confused with other conditions (e.g. stroke, depression and dementia)… Acute confusion’ or ‘impaired cognition’ are other terms that might be used to refer to delirium.” | No – there is no definition of delirium | Somewhat – there is a definition of delirium, but it uses incorrect words (e.g., disease) | Yes – delirium is accurately defined |
| 1. Does the website provide relevant background on delirium risk factors or causes of delirium? | No - there is no mention of delirium risk factors or causes | Somewhat – delirium risk factors or causes are mentioned, but may be incorrect (i.e., not supported by the available peer reviewed literature) | Yes – delirium risk factors or causes are explained in detail & are correct (i.e., supported by the available peer reviewed literature) |
| 1. Does the website provide information on short & long term outcomes of delirium? | No – there is no mention of the short & long term outcomes of delirium | Somewhat – short & long term outcomes of delirium are mentioned but are incorrect (i.e., not supported by the available peer reviewed literature) | Yes - short & long term outcomes are explained in detail & are correct (i.e., supported by the available peer reviewed literature) |
| 1. Does the education material include the signs & symptoms of delirium?   *Note:* *This is different from the definition of delirium. It should include features of delirium (e.g., inattention, fluctuating course, disorganized thinking, and altered level of consciousness) and may include delirium subtypes.* | No – there is no mention about the signs & symptoms of delirium | Somewhat – some signs & symptoms of delirium are mentioned, but hallmarks of delirium (e.g., inattention) are missing or words that are not specific to delirium (e.g., confusion) are used | Yes - signs & symptoms of delirium are mentioned (e.g., inattention, fluctuating course, disorganized thinking, altered level of consciousness) |
| 1. Does the website include information to differentiate between delirium & dementia? | No – dementia is not mentioned | Somewhat – it is stated that delirium & dementia are different, but there are no details | Yes – information about the differences between delirium & dementia are clearly presented |
| 1. Does the website include non-pharmacological delirium prevention strategies coherent with best evidence of available practice guidelines*? | No –non-pharmacological delirium prevention strategies are not mentioned | Somewhat – the non-pharmacological delirium prevention strategies are mentioned, but are not based on the most recent version clinical practice guidelines | Yes - the non-pharmacological delirium prevention strategies are mentioned and are based on the most recent version clinical practice guidelines |
| 1. Does the website present pharmacological treatment coherent with current best evidence or available practice guidelines*? | No – the education does not present pharmacological treatment coherent with best practice guidelines | Somewhat - the delirium pharmacological treatment strategies are mentioned, but are not based on the most recent version clinical practice guidelines | Yes - the delirium pharmacological treatment strategies are mentioned and are based on the most recent version clinical practice guidelines |
