# Supplementary material for: Evaluation of the Quality of Delirium Website Content for Patient and Family Education: Cross-Sectional Study
Source: J Med Internet Res. 2025 Feb 20;27:e53087. doi: 10.2196/53087 (PMC11888015; doi:10.2196/53087)
Supplement: Multimedia Appendix 4 [file jmir_v27i1e53087_app4.docx]

**Appendix 4.** Description of the websites included in the study.

*Patient engagement framework classifies materials as: INFORM (provides delirium knowledge), ACTIVATE (prompts action) or COLLABORATE (prompts interaction between public and other groups).*

| **URL** | **Website Title** | **Website Country** | **Final** | **Patient Engagement Framework** |
| --- | --- | --- | --- | --- |
| https://aci.health.nsw.gov.au/__data/assets/pdf_file/0018/181701/ACI-Aged-Delirium-brochure.pdf | Delirium | Australia | Regional health authority | inform, activate, collaborate |
| https://americandeliriumsociety.org/patients-families/what-is-delirium/ | What is Delirium? | USA | Foundation/advocacy organization | inform, activate |
| https://betterhealthwhileaging.net/hospital-delirium-what-to-do/ | Hospital Delirium: What to know & do | USA | Commercial | inform, activate |
| https://betterhealthwhileaging.net/what-is-delirium-10-things-to-know/ | 10 Things to Know About Delirium | USA | Commercial | inform, activate |
| https://cancer.ca/en/treatments/side-effects/delirium | Delirium | Canada | Foundation/advocacy organization | Inform, activate |
| https://ccsmh.ca/areas-of-focus/delirium/ | Delirium | Canada | Foundation/advocacy organization | inform, activate, collaborate |
| https://en.wikipedia.org/wiki/Delirium | Delirium | USA | General reference or educational resource | inform |
| https://www.europeandeliriumassociation.org/patient-and-carer-resources.html | Patient and Carer Resources | UK | Foundation/advocacy | inform, activate, collaborate |
| https://fluidst.ca/news/f/delirium-what-it-is-symptoms-types-what-to-do | Delirium: What It Is, Symptoms, Types & What to Do | Canada | Commercial | inform |
| https://geriatricsontario.ca/caregiving-strategies/changes-in-thinking-and-behaviour-delirium/ | Changes in thinking and behaviour (Delirium) | Canada | Government | inform, activate, collaborate |
| https://geriatricsontario.ca/resources/ontario-health-delirium-quality-standard-an-overview/ | Delirium Quality Standards – Knowledge Translation Resources | Canada | Government | inform, activate, collaborate |
| https://health.clevelandclinic.org/coronavirus-symptom-delirium | Why Delirium in Coronavirus Patients Concerns Doctors | USA | Private hospital-affiliated | inform |
| https://healthify.nz/health-a-z/d/delirium/ | Delirium | New Zealand | General reference or educational resource | inform, activate |
| https://mcpress.mayoclinic.org/healthy-brain/recognizing-and-responding-to-delirium/ | Recognizing and responding to delirium | USA | Private hospital-affiliated | inform, activate |
| https://medical-dictionary.thefreedictionary.com/delirium | Delirium | USA | General reference or educational resource | inform |
| https://medlineplus.gov/delirium.html | Delirium | USA | Government | inform |
| https://mentalhealthfoundation.org/health-conditions/cognitive-disorders/delirium/ | Delirium | UK | Foundation/advocacy organization | inform, activate, collaborate |
| https://my.clevelandclinic.org/health/diseases/15252-delirium | Delirium | USA | Private hospital-affiliated | inform, activate, collaborate |
| https://myhealth.alberta.ca/Alberta/Pages/delirium.aspx | Delirium | Canada | Government | inform, activate, collaborate |
| https://openbooks.library.baylor.edu/understandingpsychdisorders/chapter/delirium/ | Delirium | USA | Academic | inform |
| https://pakconline.com/delirium/ | Delirium | USA | Private healthcare provider | inform |
| https://patienteduc.fraserhealth.ca/file/delirium-and-nutrition-758612.pdf | Delirium and Nutrition | Canada | Regional health authority | inform, activate, collaborate |
| https://patienteduc.fraserhealth.ca/file/delirium-in-the-older-adult-a-family-guide-1311.pdf | Delirium in the Older Adult: A Family Guide | Canada | Regional health authority | inform, activate, collaborate |
| https://rgpc.ca/wp-content/uploads/2024/10/Delirium_Where-to-Start-Tool-June-19-2024.pdf | Delirium: Where to Start | Canada | Academic | inform, activate |
| https://rgptoronto.ca/wp-content/uploads/2024/04/Guidance-on-Delirium-Care-for-Older-Adults-in-the-Community-V1-2024.pdf | Guidance on Delirium Care for Older Adults in the community | Canada | Academic | inform, activate, collaborate |
| https://rnao.ca/bpg/guidelines/fact-sheets/delirium-dementia-and-depression-what-difference | Delirium, dementia and depression: What is the difference? | Canada | Professional organization | inform, collaborate |
| https://royalpapworth.nhs.uk/our-hospital/latest-news/intensive-care-delirium | Raising awareness of Intensive Care Delirium | UK | Hospital-affiliated | inform, activate, collaborate |
| https://sunnybrook.ca/content/?page=delirium | Delirium | Canada | Hospital-affiliated | inform, activate |
| https://theconversation.com/explainer-what-is-delirium-and-is-it-dangerous-67936 | Explainer: What is delirium and is it dangerous? | Australia | General reference or educational resource | inform |
| https://theconversation.com/what-is-delirium-194631 | What is Delirium? | Australia | General reference or educational resource | inform |
| https://wrha.mb.ca/2017/03/14/one-in-four-people-over-the-age-of-70-experience-delirium-in-hospital/ | One in four people over the age of 70 experience delirium in hospital | Canada | Regional health authority | inform |
| https://www.aacap.org/AACAP/Families_and_Youth/Facts_for_Families/FFF-Guide/Delirium-in-Children-and-Adolescents-120.aspx | Delirium in Children and Adolescents | USA | Professional organization | inform, activate, collaborate |
| https://www.aafp.org/pubs/afp/issues/2014/0801/p150.html | Delirium in Older Persons: Evaluation and Management | USA | Professional organization | inform, activate, collaborate |
| https://www.aarp.org/health/brain-health/global-council-on-brain-health/delirium/ | Delirium | USA | Foundation/advocacy organization | inform, activate, collaborate |
| https://www.albertahealthservices.ca/scns/Page13393.aspx | Delirium & Dementia | Canada | Regional health authority | inform, activate, collaborate |
| https://www.alzheimers.org.uk/get-support/daily-living/delirium | Delirium - symptoms, diagnosis and treatment | UK | Foundation/advocacy organization | inform, activate |
| https://www.aplaceformom.com/caregiver-resources/articles/delirium-vs-dementia | Delirium vs. Dementia: What's the difference | USA | Commercial | inform, activate, collaborate |
| https://www.ashfordstpeters.nhs.uk/delirium | Delirium | UK | Hospital-affiliated | inform |
| https://www.betterhelp.com/advice/psychosis/delirium-vs-psychosis-symptoms-similarities-and-differences/ | Delirium vs Psychosis: Symptoms, Similarities, and Differences | USA | Commercial | inform, activate, collaborate |
| https://www.betterhelp.com/mental-health/disorders-conditions/delirium/ | Delirium | USA | Commercial | inform, activate, collaborate |
| https://www.bruyere.org/en/Delirium | Delirium | Canada | Hospital-affiliated | inform, activate |
| https://www.cancer.gov/about-cancer/treatment/side-effects/delirium | Delirium: Cancer Treatment Side Effect | USA | Government | inform, activate, collaborate |
| https://www.cancer.org/cancer/managing-cancer/side-effects/changes-in-mood-or-thinking/confusion.html | Confusion and Delirium | USA | Foundation/advocacy organization | inform, activate, collaborate |
| https://www.chumontreal.qc.ca/en/fiches-sante/delirium-elderly-information-families-and-caregivers | Delirium: Information for families and caregivers | Canada | Hospital-affiliated | inform, activate, collaborate |
| https://www.cntw.nhs.uk/news/what-is-delirium-and-how-can-i-support-someone-with-it/ | What is delirium, and how can I support someone with it? | UK | Hospital-affiliated | inform |
| https://www.delirium.org.au/what-is-delirium | Delirium Insight | Australia | Foundation/advocacy organization | inform |
| https://www.deliriumcentral.org/ | Delirium Central | USA | Academic | inform, activate, collaborate |
| https://www.dementia.org.au/living-dementia/mood-and-behaviour-changes/delirium | Delirium | Australia | Foundation/advocacy organization | inform, activate |
| https://www.dementiauk.org/information-and-support/health-advice/delirium/ | Delirium (sudden confusion) | UK | Foundation/advocacy organization | inform, activate |
| https://www.ementalhealth.ca/Canada/Delirium-Information-for-Parents-and-Caregivers/index.php?m=article&ID=24408 | Delirium: Information for families and caregivers | Canada | Hospital-affiliated | inform, activate |
| https://www.fraserhealth.ca/health-topics-a-to-z/seniors/delirium | Delirium | Canada | Regional health authority | inform, activate, collaborate |
| https://www.guysandstthomas.nhs.uk/health-information/delirium-sudden-confusion | Delirium (sudden confusion) | UK | Hospital-affiliated | inform, activate |
| https://www.haltonhealthcare.on.ca/site_Files/Content/pdfs/01_Patients/02_Patient-Safety/What_is_Delirium.pdf | What is Delirium? | Canada | Regional health authority | inform, activate, collaborate |
| https://www.hamiltonhealthsciences.ca/share/preventing-delirium-in-the-hospital/ | Preventing delirium in the hospital | Canada | Regional health authority | inform, activate, collaborate |
| https://www.health.harvard.edu/blog/the-dangers-of-hospital-delirium-in-older-people-201111163810 | The dangers of hospital delirium in older people | USA | Academic | inform, activate, collaborate |
| https://www.healthdirect.gov.au/delirium | Delirium | Australia | Government | inform, activate |
| https://www.healthhub.sg/a-z/diseases-and-conditions/delirium | Delirium: Symptoms and Management | Singapore | Government | inform, activate |
| https://www.healthinaging.org/a-z-topic/delirium | Delirium | USA | Foundation/advocacy organization | inform, activate, collaborate |
| https://www.healthline.com/health/delirium | What’s Delirium and How Does It Happen? | USA | Commercial | inform, activate, collaborate |
| https://www.healthline.com/health/dementia/delirium-vs-dementia | Is Delirium Different from Dementia? | USA | Commercial | inform, activate, collaborate |
| https://www.healthline.com/health/hospital-delirium | What Every Caregiver Should Know About Hospital Delirium | USA | Commercial | inform, activate |
| https://www.hopkinsmedicine.org/health/conditions-and-diseases/delirium | Delirium | USA | Academic | inform, activate, collaborate |
| https://www.hqontario.ca/Portals/0/documents/evidence/quality-standards/qs-delirium-patient-guide-en.pdf | Delirium  Suggestions on what to discuss with the health care team to help your family member receive high-quality care | Canada | Government | inform, activate, collaborate |
| https://www.hrh.ca/patient-education/005031_Delirium.pdf | Delirium  Information for Patients, Families, and Friends | Canada | Hospital-affiliated | inform, activate, collaborate |
| https://www.hse.ie/eng/dementia-pathways/files/delirium-information-leaflet.pdf | What is delirium? | Ireland | Foundation/advocacy organization | inform, activate |
| https://www.icudelirium.org/patients-and-families/overview | Patients and Families Overview Critical Illness, Brain Dysfunction, and Survivorship (CIBS) Center | USA | Foundation/advocacy organization | inform, activate, collaborate |
| https://www.interiorhealth.ca/stories/aging-better-know-the-difference-between-delirium-and-dementia | Aging Better: Know the difference between delirium and dementia | Canada | Regional health authority | inform, activate, collaborate |
| https://www.interiorhealth.ca/stories/why-delirium-should-be-treated-medical-emergency | Why delirium should be treated as a medical emergency | Canada | Regional health authority | inform, activate, collaborate |
| https://www.islandhealth.ca/learn-about-health/seniors/delirium | Delirium | Canada | Regional health authority | inform |
| https://www.islandhealth.ca/sites/default/files/seniors/documents/delirium-patient-family-handout.pdf | Delirium | Canada | Regional health authority | inform, activate, collaborate |
| https://www.lavalensante.com/fileadmin/internet/cisss_laval/Soins_et_services/Geriatrie_-_PRAG/DeliriumANG.pdf | Major Neurocognitive disorders: Dementia | Canada | Government | inform, activate, collaborate |
| https://www.mariecurie.org.uk/professionals/palliative-care-knowledge-zone/symptom-control/delirium | Delirium when someone has a terminal illness | United Kingdom | Foundation/advocacy organization | inform, activate, collaborate |
| https://www.mayoclinic.org/diseases-conditions/delirium/diagnosis-treatment/drc-20371391 | Delirium | USA | Private hospital-affiliated | inform, activate |
| https://www.mayoclinichealthsystem.org/hometown-health/speaking-of-health/recognizing-and-responding-to-delirium | Recognizing and responding to delirium | USA | Private hospital-affiliated | inform, activate |
| https://www.medicalnewstoday.com/articles/326684 | What to know about delirium | USA | Commercial | inform, activate, collaborate |
| https://www.mentalhealth.com/library/delirium | Delirium | UK | Commercial | Inform, activate |
| https://www.merckmanuals.com/en-ca/home/brain-spinal-cord-and-nerve-disorders/delirium-and-dementia/delirium | Delirium | USA | Commercial | inform, activate, collaborate |
| https://www.mountsinai.org/health-library/diseases-conditions/delirium | Delirium | USA | Private hospital-affiliated | inform, activate, collaborate |
| https://www.mskcc.org/cancer-care/patient-education/delirium | Caring for Someone With Delirium | USA | Private hospital-affiliated | inform, activate, collaborate |
| https://www.nationwidechildrens.org/conditions/delirium | Delirium | USA | Private hospital-affiliated | inform, activate, collaborate |
| https://www.nbt.nhs.uk/patients-carers/coming-hospital/dementia-care/patient-information-delirium | Patient information-Delirium | United Kingdom | Government | inform, activate, collaborate |
| https://www.news-medical.net/health/Delirium-Symptoms.aspx | Delirium symptoms | United Kingdom | Commercial | inform |
| https://www.news-medical.net/health/What-is-Delirium.aspx | What is delirium? | United Kingdom | Commercial | inform |
| https://www.nhs.uk/conditions/confusion/ | Sudden confusion (delirium) | United Kingdom | Government | inform, activate, collaborate |
| https://www.nhsinform.scot/delirium | Delirium | United Kingdom | Government | Inform, activate |
| https://www.nidirect.gov.uk/conditions/sudden-confusion-delirium | Sudden confusion (delirium) | United Kingdom | Government | inform, activate, collaborate |
| https://www.nshealth.ca/frailty/delirium | Delirium | Canada | Regional health authority | inform, activate, collaborate |
| https://www.nslhd.health.nsw.gov.au/delirium/Pages/what-is-delirium.aspx | What is delirium? | Australia | Regional health authority | inform |
| https://www.nth.nhs.uk/resources/delirium/ | Delirium | United Kingdom | Government | inform, activate, collaborate |
| https://www.prhc.on.ca/wp-content/uploads/2020/02/Patient-Information-Delirium-Brochure.pdf | Delirium: A guide for patients, family members, and caregivers | Canada | Government | inform, activate, collaborate |
| https://www.psychologytoday.com/ca/conditions/delirium | Delirium | USA | Commercial | inform, activate, collaborate |
| https://www.qch.on.ca/DeliriumPreventionandManagement | Delirium Prevention and Management | Canada | Hospital-affiliated | inform, activate |
| https://www.rcpsych.ac.uk/mental-health/mental-illnesses-and-mental-health-problems/delirium | Delirium | United Kingdom | Professional organization | inform, activate |
| https://www.sbm.org/healthy-living/caring-for-an-older-adult-how-to-detect-delirium-in-your-loved-one-and-what-you-can-do | Caring for an Older Adult? How to Detect Delirium in Your Loved One, and What You Can Do | USA | Foundation/advocacy organization | inform, activate, collaborate |
| https://www.sign.ac.uk/assets/pat157.pdf | Delirium: A booklet for people who have experienced delirium, and for their carers | UK | Government | inform, activate, collaborate |
| https://www.smgh.ca/areas-of-care/cardiac-care/delirium-booklet_aoda.pdf | On the lookout for delirium: a guide for patients, families, and friends | Canada | Hospital-affiliated | inform, activate, collaborate |
| https://www.uhcw.nhs.uk/download/clientfiles/files/Patient%20Information%20Leaflets/Medicine/Care%20of%20the%20Elderly/Delirium.pdf | Care of the Elderly, Delirium: Information for relatives, carers, and patients | UK | Government | inform, activate, collaborate |
| https://www.uhn.ca/patientsfamilies/health_information/health_topics/documents/delirium.pdf | Delirium: Information for patients, families, friends and caregivers | Canada | Hospital-affiliated | inform, activate, collaborate |
| https://www.uhsussex.nhs.uk/resources/recognising-delirium/ | Recognising delirium: Information about the symptoms and treatment for delirium which is a state of mental confusion | UK | Government | inform, activate, collaborate |
| https://www.uptodate.com/contents/delirium-beyond-the-basics | Patient education: Delirium (Beyond the Basics) | USA | Commercial | inform, activate, collaborate |
| https://www.va.gov/GERIATRICS/pages/delirium_topics.asp | Geriatrics and Extended Care | USA | Government | inform, activate, collaborate |
| https://www.verywellhealth.com/delirium-5223127 | Delirium | USA | Commercial | inform, activate, collaborate |
| https://www.verywellhealth.com/delirium-and-terminal-restlessness-1132475 | Terminal Restlessness and Delirium at the End of Life: Common Characteristics | USA | Commercial | inform, activate, collaborate |
| https://www.verywellhealth.com/delirium-what-you-should-know-3156864 | Delirium: What you should know: Causes, Risk Factors, Diagnosis, and Treatment | USA | Commercial | inform, activate, collaborate |
| https://www.webmd.com/brain/sudden-confusion-causes | Conditions That Cause Sudden Confusion | USA | Commercial | inform, activate |
| https://www.wikihow.health/Care-for-Someone-with-Delirium | How to care for someone with delirium | USA | General Reference or Educational Resource | inform, activate |
